# Draft Genome Sequence of Pseudomonas nitrititolerans Strain AGROB37, Isolated from a Sheep Dairy Farm in New Zealand

**DOI:** 10.1128/MRA.00564-20

**Published:** 2020-07-09

**Authors:** Tanushree B. Gupta, Ruy Jauregui, Alexis N. Risson, Gale Brightwell, Paul Maclean

**Affiliations:** a Food Assurance Team, Hopkirk Research Institute, AgResearch, Palmerston North, New Zealand; b Knowledge and Analytics, Grasslands Research Centre, AgResearch, Palmerston North, New Zealand; University of Arizona

## Abstract

We report the draft genome sequence of a new Pseudomonas nitrititolerans strain, AGROB37, isolated from a sheep dairy farm environment in New Zealand. The genome is 4.19 Mbp long, with a GC content of 63.2%. The genome sequence was found to be closely related to that of the type strain *Pseudomonas nitrititolerans* GL14.

## ANNOUNCEMENT

The genus *Pseudomonas*, first proposed by Migula in 1894 (1), consists of about 254 species of Gram-negative, rod-shaped, non-spore-forming bacteria. These bacteria are usually aerobic, but a few have been found to be facultative anaerobes (2–4). *Pseudomonas* species are commonly present in environments such as soil, water, and plants and can also be isolated from human skin and throat (5–9). While some *Pseudomonas* species, such as Pseudomonas aeruginosa and P. syringae, are associated with biofilm formation and human, animal, and plant pathogenicity, other species, such as P. alcaligenes, P. putida, and P. stutzeri, have been applied as bioremediation agents (9–14).

A new species of *Pseudomonas*, *P. nitrititolerans* (type strain GL14), was recently isolated from a nitrification/denitrification bioreactor in a laboratory based in Beijing, China. This species was found to be facultatively anaerobic, to utilize sodium nitrite as a sole nitrogen source, and to be highly nitrite tolerant (4).

In the present study, we report the whole-genome sequence of a new *Pseudomonas nitrititolerans* strain, AGROB37, isolated from a woodchip bedding sample collected from a New Zealand sheep dairy farm. The sequences obtained will be used to investigate pathogenic or beneficial traits of the new strain.

Samples were processed using the methodology, with slight modifications, described in reference 15. Briefly, 25 g of woodchip bedding material was weighed in a stomacher bag and suspended in 100 ml of phosphate buffer (PB). The suspended sample was blended well and centrifuged at 3,466 × *g* for 1 h. The pellet was resuspended in 25 ml of PB, and 1 ml of the suspension was 10-fold serially diluted and plated onto cetrimide-fucidin-cephalosporin (CFC) agar plates to isolate *Pseudomonas* strain AGROB37 (16). Genomic DNA was extracted from these pure cultures grown in tryptic soy broth (Fort Richard, New Zealand) using the phenol-chloroform extraction method (17). Quality and concentration of DNA were determined using a Qubit 2.0 fluorometer (Thermo Fisher Scientific, USA).

The whole-genome sequence of *Pseudomonas* species strain AGROB37 was prepared via the NuGEN Celero DNA enzymatic library kit and sequenced using the Illumina MiSeq sequencing platform version 3 (Massey Genome Services, Palmerston North, New Zealand) to produce 452,698 paired-end reads of 301 bp, giving a coverage of roughly 65-fold. The reads were quality trimmed, filtered, and assembled via the A5-miseq pipeline version 20160825 with default settings (18). The assembly produced 43 contigs with a total genome size of 4.2 Mb, an *N*_50_ value of 226 kb, and a GC content of 63.2%. A BUSCO version 3.0.2 (19) test using the bacterial reference produced a completeness score of 100%.

A two-way average nucleotide identity test (http://enve-omics.ce.gatech.edu/ani/) of the new *Pseudomonas* strain AGROB37 produced a 98% value matching with Pseudomonas nitrititolerans GL14^T^ (20). Furthermore, a comparative genomic analysis was performed with the genome sequences of these organisms using *in silico* DNA-DNA hybridization (dDDH) via the Type (strain) Genome Server (TYGS) (https://tygs.dsmz.de/) (21). This analysis resulted in a dDDH (d_6_) value of 89.6%, indicating the same species but with probable differences at the strain level. Further studies are required to investigate these differences.

As part of the submission process, NCBI annotated the genomic scaffolds with Prokaryotic Genome Annotation Pipeline (PGAP) (22), resulting in 4,003 genes being annotated in total.

### Data availability.

This whole-genome shotgun project has been deposited in DDBJ/ENA/GenBank under the accession number JABEVZ000000000. The version described in this paper is version JABEVZ010000000. The raw sequencing data have been deposited in the SRA under the accession number SRR11665885 and BioProject accession number PRJNA629605.
